# Supplementary material for: COVID-19 Mortality in the Colorado Center for Personalized Medicine Biobank
Source: Int J Environ Res Public Health. 2023 Jan 29;20(3):2368. doi: 10.3390/ijerph20032368 (PMC9916246; doi:10.3390/ijerph20032368)
Supplement: Supplementary file 1 [file ijerph-20-02368-s001.zip › Table S1. Phecodes.pdf]

Table S1: Phecodes. All the phecodes used throughout the study are listed by phenotype category.

Phenotype Category		Phecode	Phenotype	Deaths from COVID-19	Total in Biobank
Diabetes		250	Diabetes mellitus	68	15,836
		250.1	Type 1 diabetes	<10	1,930
		250.11	Type 1 diabetes with ketoacidosis	<10	133
		250.12	Type 1 diabetes with renal manifestations	<10	334
		250.13	Type 1 diabetes with ophthalmic manifestations	<10	276
		250.14	Type 1 diabetes with neurological manifestations	<10	406
		250.15	Diabetes type 1 with peripheral circulatory disorders	<10	41
		250.2	Type 2 diabetes	67	14,795
		250.21	Type 2 diabetes with ketoacidosis	<10	53
		250.22	Type 2 diabetes with renal manifestations	30	1,956
		250.23	Type 2 diabetes with ophthalmic manifestations	<10	909
		250.24	Type 2 diabetes with neurological manifestations	18	2,767
		250.25	Diabetes type 2 with peripheral circulatory disorders	<10	394
		250.3	Insulin pump user	18	3,623
		250.4	Abnormal glucose	12	6,636
		250.41	Impaired fasting glucose	<10	2,099
		250.42	Other abnormal glucose	<10	4,195
		250.5	Glycosuria or Acetonuria	<10	27
		250.6	Polyneuropathy in diabetes	12	1,714
		250.7	Diabetic retinopathy	<10	684
Control Phecodes	Mental Disorders	292	Neurological disorders	26	7,037
		296	Mood disorders	48	23,486
		296.2	Depression	43	19,561
		296.22	Major depressive disorder	23	10,785
		300	Anxiety disorders	25	27,266
		300.1	Anxiety disorder	24	23,431
	Neurological	327	Sleep disorders	30	16,665
		327.3	Sleep apnea	51	20,282
		327.32	Obstructive sleep apnea	36	14,682
		327.4	Insomnia	15	11,068
	Sense Organs (Eyes)	366	Cataract	30	8,062
		366.2	Senile cataract	20	5,417
Cardiovascular		394	Rheumatic disease of the heart valves	10	1,328
		394.1	Mitral valve stenosis and aortic valve stenosis	<10	45

	394.2	Mitral valve disease	<10	398
	394.3	Aortic valve disease	<10	21
	394.4	Acute rheumatic heart disease	<10	19
	394.7	Disease of tricuspid valve	<10	324
	395	Heart valve disorders	22	4,302
	395.1	Nonrheumatic mitral valve disorders	10	1,604
	395.2	Nonrheumatic aortic valve disorders	<10	1,965
	395.3	Nonrheumatic tricuspid valve disorders	<10	130
	395.4	Nonrheumatic pulmonary valve disorders	<10	106
	395.6	Heart valve replaced	<10	1,099
	396	Abnormal heart sounds	<10	1,387
	401	Hypertension	123	40,020
	401.1	Essential hypertension	109	37,968
	401.2	Hypertensive heart and/or renal disease	56	4,374
	401.21	Hypertensive heart disease	<10	562
	401.22	Hypertensive chronic kidney disease	37	2,376
	401.3	Other hypertensive complications	<10	644
	402	Elevated blood pressure reading without diagnosis of hypertension	<10	<10
	411	Ischemic Heart Disease	41	10,004
	411.1	Unstable angina (intermediate coronary syndrome)	<10	400
	411.2	Myocardial infarction	13	3,065
	411.3	Angina pectoris	<10	1,124
	411.4	Coronary atherosclerosis	32	8,816
	411.41	Aneurysm and dissection of heart	<10	107
	411.8	Other chronic ischemic heart disease, unspecified	<10	742
	411.9	Other acute and subacute forms of ischemic heart disease	<10	14
	414	Other forms of chronic heart disease	<10	789
	414.2	ASCVD	<10	<10
	415	Pulmonary heart disease	25	4,370
	415.1	Acute pulmonary heart disease	<10	2,093
	415.11	Pulmonary embolism and infarction, acute	<10	2,038
	415.2	Chronic pulmonary heart disease	13	1,296
	415.21	Primary pulmonary hypertension	<10	122
	416	Cardiomegaly	<10	933
	418	Nonspecific chest pain	27	10,587
	418.1	Precordial pain	<10	778
	420	Carditis	<10	860
	420.1	Myocarditis	<10	39
	420.2	Pericarditis	<10	593
	420.21	Acute pericarditis	<10	124
	420.22	Chronic pericarditis	<10	34
	420.3	Endocarditis	<10	220
	425	Cardiomyopathy	11	2,270
	425.1	Primary/intrinsic cardiomyopathies	11	2,020
	425.11	Hypertrophic obstructive cardiomyopathy	<10	134

	425.12	Other hypertrophic cardiomyopathy	<10	171
	425.2	Secondary/extrinsic cardiomyopathies	<10	145
	425.8	Other cardiomyopathy	<10	111
	426	Cardiac conduction disorders	46	8,411
	426.2	Atrioventricular [AV] block	<10	1,004
	426.21	First degree AV block	<10	211
	426.22	Mobitz II AV block	<10	<10
	426.23	Second degree AV block	<10	117
	426.24	Atrioventricular block, complete	<10	273
	426.25	Other heart block	<10	41
	426.3	Bundle branch block	12	1,932
	426.31	Right bundle branch block	<10	729
	426.32	Left bundle branch block	<10	685
	426.4	Anomalous atrioventricular excitation	<10	126
	426.7	Abnormal electrocardiogram [ECG] [EKG]	14	2,413
	426.8	Other cardiac conduction disorders	<10	171
	426.9	Cardiac pacemaker/device in situ	<10	2,637
	426.91	Cardiac pacemaker in situ	<10	1,768
	426.92	Cardiac defibrillator in situ	<10	948
	427	Cardiac dysrhythmias	61	19,106
	427.1	Paroxysmal tachycardia, unspecified	<10	1,878
	427.11	Paroxysmal supraventricular tachycardia	<10	685
	427.12	Paroxysmal ventricular tachycardia	<10	779
	427.2	Atrial fibrillation and flutter	32	5,959
	427.21	Atrial fibrillation	29	5,527
	427.22	Atrial flutter	<10	1,405
	427.3	Other specified cardiac dysrhythmias	11	2,898
	427.4	Cardiac arrest and ventricular fibrillation	<10	359
	427.41	Ventricular fibrillation and flutter	<10	103
	427.42	Cardiac arrest	<10	269
	427.5	Arrhythmia (cardiac) NOS	<10	451
	427.6	Premature beats	<10	2,379
	427.61	Supraventricular premature beats	<10	360
	427.7	Tachycardia NOS	<10	1,745
	427.8	Sinoatrial node dysfunction (Bradycardia)	<10	536
	427.9	Palpitations	<10	4,468
	428	Congestive heart failure; nonhypertensive	31	4,183
	428.1	Congestive heart failure (CHF) NOS	16	2,106
	428.2	Heart failure NOS	<10	56
	428.3	Heart failure with reduced EF [Systolic or combined heart failure]	16	2,046
	428.4	Heart failure with preserved EF [Diastolic heart failure]	14	1,155
	429	Ill-defined descriptions and complications of heart disease	<10	922
	429.1	Heart transplant/surgery	<10	290
	429.2	Abnormal function study of cardiovascular system	<10	396
	429.3	Symptoms involving cardiovascular system	<10	254

	429.9	Cardiac complications, not elsewhere classified	<10	23
	430	Intracranial hemorrhage	<10	635
	430.1	Subarachnoid hemorrhage	<10	181
	430.2	Intracerebral hemorrhage	<10	202
	430.3	Subdural hemorrhage	<10	181
	433	Cerebrovascular disease	36	6,496
	433.1	Occlusion and stenosis of precerebral arteries	10	1,762
	433.11	Occlusion of cerebral arteries, with cerebral infarction	<10	85
	433.12	Cerebral atherosclerosis	<10	<10
	433.2	Occlusion of cerebral arteries	15	1,457
	433.21	Cerebral artery occlusion, with cerebral infarction	14	1,418
	433.3	Cerebral ischemia	22	3,920
	433.31	Transient cerebral ischemia	21	3,869
	433.32	Moyamoya disease	<10	17
	433.5	Cerebral aneurysm	<10	353
	433.6	Acute, but ill-defined cerebrovascular disease	<10	<10
	433.8	Late effects of cerebrovascular disease	<10	672
	440	Atherosclerosis	<10	1,191
	440.1	Atherosclerosis of renal artery	<10	130
	440.2	Atherosclerosis of the extremities	<10	404
	440.21	Atherosclerosis of native arteries of the extremities with ulceration or gangrene	<10	60
	440.22	Atherosclerosis of native arteries of the extremities with intermittent claudication	<10	203
	440.9	Atherosclerosis of aorta	<10	302
	441	Vascular insufficiency of intestine	<10	162
	441.1	Acute vascular insufficiency of intestine	<10	11
	441.2	Chronic vascular insufficiency of intestine	<10	57
	442	Other aneurysm	<10	2,129
	442.1	Aortic aneurysm	<10	1,664
	442.11	Abdominal aortic aneurysm	<10	503
	442.2	Aneurysm of iliac artery	<10	95
	442.3	Aneurysm of artery of lower extremity	<10	46
	442.4	Arterial dissection	<10	163
	442.8	Aneurysm of other specified artery	<10	158
	443	Peripheral vascular disease	11	2,312
	443.1	Raynaud's syndrome	<10	923
	443.7	Peripheral angiopathy in diseases classified elsewhere	<10	164
	443.8	Other specified peripheral vascular diseases	<10	31
	443.9	Peripheral vascular disease, unspecified	<10	1,137
	444	Arterial embolism and thrombosis	<10	257
	444.1	Arterial embolism and thrombosis of lower extremity artery	<10	48
	444.2	Arterial embolism and thrombosis	<10	21
	444.5	Atheroembolism	<10	15
	446	Polyarteritis nodosa and allied conditions	<10	416

	446.1	Thromboangiitis obliterans	<10	14
	446.2	Acute febrile mucocutaneous lymph node syndrome (Kawasaki disease)	<10	<10
	446.3	Hypersensitivity angiitis	<10	34
	446.4	Wegener's granulomatosis	<10	119
	446.5	Giant cell arteritis	<10	74
	446.6	Polyarteritis nodosa	<10	35
	446.7	Takayasu's disease	<10	<10
	446.8	Thrombotic microangiopathy	<10	20
	446.9	Arteritis NOS	<10	150
	447	Other disorders of arteries and arterioles	<10	1,933
	447.1	Stricture of artery	<10	110
	447.7	Aortic ectasia	<10	985
	448	Disease of capillaries	<10	54
	450	Noninfectious disorders of lymphatic channels	<10	1,344
	451	Phlebitis and thrombophlebitis	<10	250
	451.2	Phlebitis and thrombophlebitis of lower extremities	<10	99
	452	Other venous embolism and thrombosis	24	5,626
	452.1	Iatrogenic pulmonary embolism and infarction	<10	<10
	452.2	Deep vein thrombosis [DVT]	18	3,062
	452.8	Postphlebitic syndrome	<10	55
	453	Chronic venous hypertension	<10	188
	454	Varicose veins	<10	1,524
	454.1	Varicose veins of lower extremity	<10	1,345
	454.11	Varicose veins of lower extremity, symptomtic	<10	830
	455	Hemorrhoids	11	3,105
	456	Chronic venous insufficiency [CVI]	<10	1,001
	457	Encounter for long-term (current) use of anticoagulants, antithrombotics, aspirin	23	3,666
	457.2	Encounter for long-term (current) use of antiplatelets/antithrombotics	<10	195
	457.3	Encounter for long-term (current) use of aspirin	13	1,783
	458	Hypotension	14	2,165
	458.1	Orthostatic hypotension	<10	652
	458.2	Iatrogenic hypotension	<10	42
	458.9	Hypotension NOS	<10	975
	459	Other disorders of circulatory system	<10	2,651
	459.1	Hemorrhage NOS	<10	48
	459.7	Blood vessel replaced	<10	232
	459.9	Circulatory disease NEC	<10	1,589
	464	Acute sinusitis	<10	3,885
Respiratory	465	Acute upper respiratory infections of multiple or unspecified sites	14	9,614
	465.2	Acute pharyngitis	<10	2,700
	465.4	Acute laryngitis and tracheitis	<10	99
	470	Septal Deviations/Turbinate Hypertrophy	<10	1,552
	471	Nasal polyps	<10	435
	472	Chronic pharyngitis and nasopharyngitis	<10	1,171

	473	Diseases of the larynx and vocal cords	<10	2,760
	473.1	Chronic laryngitis	<10	43
	473.3	Paralysis/spasm of vocal cords or larynx	<10	454
	473.4	Voice disturbance	<10	1,703
	474	Acute and chronic tonsillitis	<10	650
	474.1	Acute tonsillitis	<10	122
	474.2	Chronic tonsillitis and adenoiditis	<10	465
	475	Chronic sinusitis	11	4,053
	475.9	Postnasal drip	<10	312
	476	Allergic rhinitis	<10	7,524
	477	Epistaxis or throat hemorrhage	<10	585
	478	Throat pain	<10	87
	479	Other upper respiratory disease	<10	3,055
	480	Pneumonia	29	3,265
	480.1	Bacterial pneumonia	<10	300
	480.12	Pseudomonal pneumonia	<10	15
	480.13	MRSA pneumonia	<10	<10
	480.2	Viral pneumonia	<10	119
	480.3	Pneumonia due to fungus (mycoses)	<10	64
	480.5	Bronchopneumonia and lung abscess	<10	40
	481	Influenza	<10	382
	483	Acute bronchitis and bronchiolitis	<10	822
	495	Asthma	21	14,689
	495.1	Chronic obstructive asthma	<10	24
	495.11	Chronic obstructive asthma with exacerbation	<10	<10
	495.2	Asthma with exacerbation	<10	2,435
	496	Chronic airway obstruction	36	5,228
	496.1	Emphysema	14	1,381
	496.2	Chronic bronchitis	16	1,289
	496.21	Obstructive chronic bronchitis	10	743
	496.3	Bronchiectasis	<10	289
	497	Bronchitis	<10	684
	498	Acute bronchospasm	<10	101
	499	Cystic fibrosis	<10	64
	500	Lung disease due to external agents	<10	202
	500.1	Extrinsic allergic alveolitis	<10	71
	500.2	Pneumoconiosis	<10	12
	501	Pneumonitis due to inhalation of food or vomitus	<10	138
	502	Postinflammatory pulmonary fibrosis	<10	389
	503	Pulmonary congestion and hypostasis	<10	43
	504	Other alveolar and parietoalveolar pneumonopathy	<10	351
	504.1	Idiopathic fibrosing alveolitis	<10	114
	505	Other pulmonary inflamation or edema	<10	88
	506	Empyema and pneumothorax	<10	333
	507	Pleurisy; pleural effusion	<10	1,246
	508	Pulmonary collapse; interstitial and compensatory emphysema	<10	192
	509	Respiratory failure, insufficiency, arrest	56	4,683

	509.1	Respiratory failure	38	2,710
	509.2	Respiratory insufficiency	<10	<10
	509.3	Pulmonary insufficiency or respiratory failure following trauma and surgery	<10	15
	509.5	Respiratory arrest	<10	<10
	509.8	Dependence on respirator [Ventilator] or supplemental oxygen	16	1,235
	510	Other diseases of lung	<10	591
	510.2	Lung transplant	<10	182
	512	Other symptoms of respiratory system	78	22,354
	512.1	Wheezing	<10	266
	512.2	Painful respiration	<10	371
	512.3	Abnormal chest sounds	<10	<10
	512.7	Shortness of breath	43	8,091
	512.8	Cough	19	5,243
	512.9	Other dyspnea	34	5,879
	513	Respiratory abnormalities	<10	614
	513.3	Hypoventilation	<10	378
	513.31	Apnea	<10	119
	513.32	Orthopnea	<10	38
	513.4	Hyperventilation	<10	13
	513.8	Disorders of diaphragm	<10	174
	514	Abnormal findings examination of lungs	<10	1,596
	514.1	Abnormal results of function study of pulmonary system	<10	67
	514.2	Solitary pulmonary nodule	<10	2,114
	516	Abnormal sputum	<10	205
	516.1	Hemoptysis	<10	195
	519	Other diseases of respiratory system, not elsewhere classified	<10	668
	519.1	Tracheostomy complications	<10	23
	519.2	Respiratory complications	<10	<10
	519.8	Other diseases of respiratory system, NEC	<10	391
	519.9	Symptoms involving respiratory system and other chest symptoms	<10	152

*Green phecodes are those with at least 10 COVID-19 deaths and were selected for additional analysis
