# Supplementary figures and images for: COVID-19 Mortality in the Colorado Center for Personalized Medicine Biobank
Source: Int J Environ Res Public Health. 2023 Jan 29;20(3):2368. doi: 10.3390/ijerph20032368 (PMC9916246; doi:10.3390/ijerph20032368)

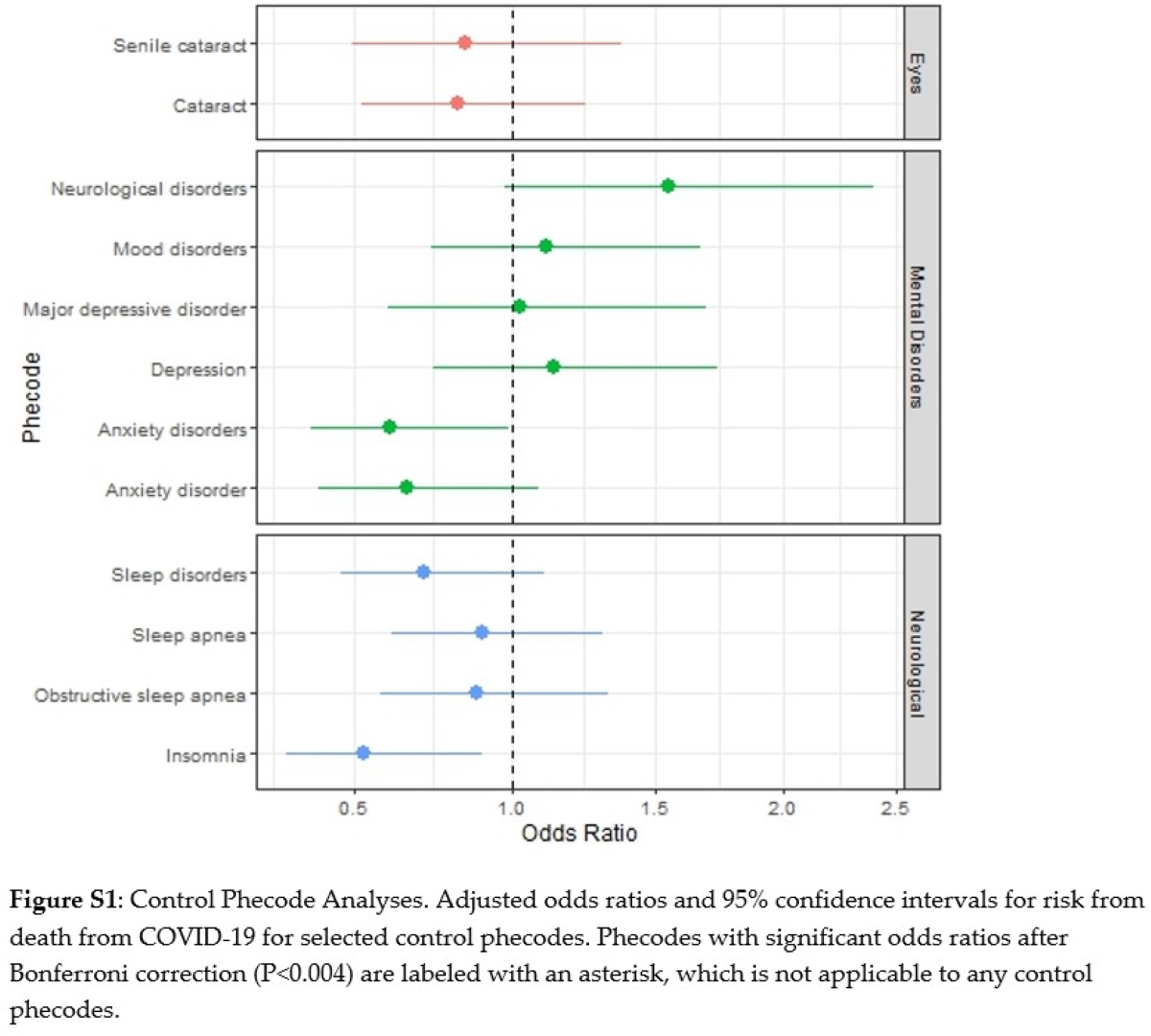

Supplement: Supplementary file 1 [file ijerph-20-02368-s001.zip › Figure S1. Control Phecode Analyses.png]

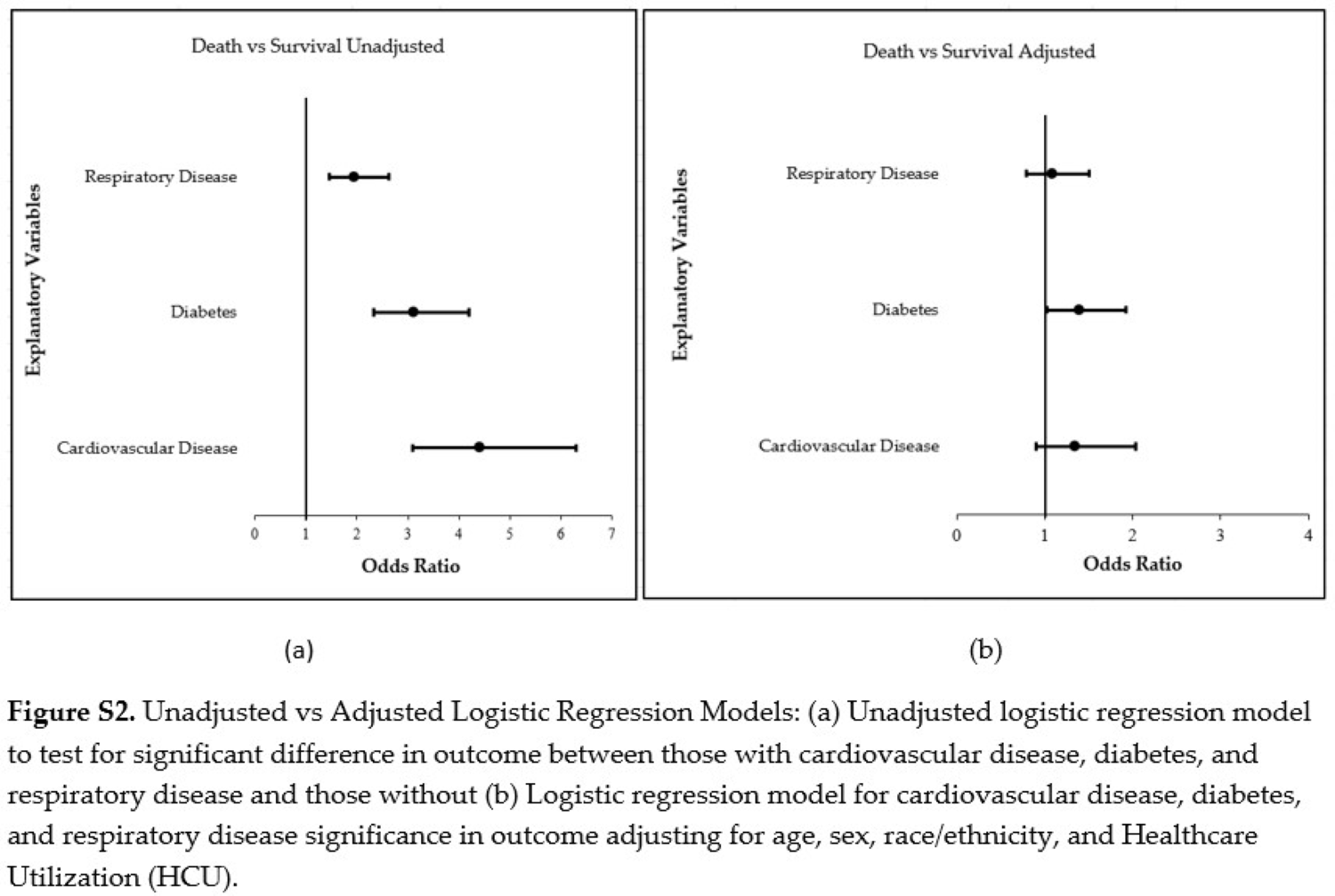

Supplement: Supplementary file 1 [file ijerph-20-02368-s001.zip › Figure S2. Unadjusted vs Adjusted Logistic Regression Models.png]
